# Cost-utility and budget impact analysis of neoadjuvant dual HER2 targeted therapy for HER2-positive breast cancer in Sri Lanka

**DOI:** 10.1038/s41598-024-67598-2

**Published:** 2024-07-20

**Authors:** Agampodi Danushi Mendis Gunasekara, Sitaporn Youngkong, Thunyarat Anothaisintawee, Thitiya Dejthevaporn, Rohini Fernandopulle, Usa Chaikledkaew

**Affiliations:** 1 Mahidol University Health Technology Assessment (MUHTA) Graduate Program, Mahidol University, Bangkok, Thailand; 2 Department of Paraclinical Sciences, Faculty of Medicine, General Sir John Kotelawala Defence University, Ratmalana, Sri Lanka; 3 Social and Administrative Pharmacy Division, Department of Pharmacy, Faculty of Pharmacy, Mahidol University, 447 Sri-Ayudhaya Rd., Phayathai, Ratchathevi, Bangkok, Thailand; 4 Department of Family Medicine, Faculty of Medicine, Ramathibodi Hospital, Mahidol University, Bangkok, Thailand; 5 Division of Medical Oncology, Ramathibodi Hospital, Mahidol University, Bangkok, Thailand

**Keywords:** Cost-utility analysis, Neoadjuvant, HER2 positive breast cancer, Targeted therapy, Breast cancer, Health care economics

## Abstract

This study aimed to assess the cost-utility and budget impact of dual to single HER2 targeted neoadjuvant therapy for HER2-positive breast cancer in Sri Lanka. A five-health state Markov model with lifetime horizon was used to assess the cost-utility of neoadjuvant trastuzumab (T) plus pertuzumab (P) or lapatinib (L) compared to single therapy of T with chemotherapy (C), in public healthcare system and societal perspectives. Input parameters were estimated using local data, network meta-analysis, published reports and literature. Costs were adjusted to year 2021 (1USD = LKR194.78). Five-year budget impact for public healthcare system was assessed. Incremental cost-effectiveness ratios in societal perspective for neoadjuvantLTC plus adjuvantT (strategy 3), neoadjuvantPTC plus adjuvantT (strategy 2), neoadjuvantLTC plus adjuvantLT (strategy 5), and neoadjuvantPTC plus adjuvantPT (strategy 4) compared to neoadjuvantTC plus adjuvantT (strategy 1) were USD2716, USD5600, USD6878, and USD12127 per QALY gained, respectively. One GDP per-capita (USD3815) was considered as the cost-effectiveness threshold for the analysis. Even though only the ICER for strategy 3 was cost-effective, uncertainty of efficacy parameter was revealed. For strategy 2 neoadjuvant PTC plus adjuvant T, a 25% reduction of neoadjuvant regimen cost was required to be cost effective for use in early HER2 positive breast cancer.

## Introduction

Breast cancer has been recognized as the most common type of cancer among females and resulted in more than 685,000 deaths in 2020 worldwide^1,2^. It is also the leading cause of cancer deaths and cause of 13.4% of total new cancer cases in 2020 in Sri Lanka^3,4^. Human epidermal growth factor receptor-2 (HER2) positivity which results in high recurrence rates, increased risk of metastasis and poorer outcomes for patients^5^, is reported to be around 15–20% among all breast cancers^6,7^. While the burden of the disease is high, challenges exist in deciding treatments such as HER2-targeted medicines which are main-stay in treatment of HER2-positive breast cancer, and making them timely available, accessible and affordable for patients. In early to locally advanced stages of breast cancer, neoadjuvant (preoperative) therapy with HER2-targeted medicines has shown to increase the chance of achieving pathological complete response (pCR) as well as improve survival^8–13^. Clinical trials have found that the addition of the single HER2-targeted medicine trastuzumab to chemotherapy in neoadjuvant treatment significantly improved pCR rate as well as disease free survival^14^. Neoadjuvant treatment with dual HER2-targeted therapy of trastuzumab and pertuzumab provided further benefits to HER2-positive patients^13^ including those with hormone receptor (HR) negativity^15^. Combinations with other HER2-targeted medicines such as tyrosine kinase inhibitors (i.e., lapatinib, neratinib) and trastuzumab emtansine (T-DM1) have also showed an increase in pCR compared to single HER2-targeted therapy^16–19^. Moreover, subsequent meta-analyses^20–22^ have found that the dual HER2-targeted therapy tended to be superior to single therapy. Furthermore, increased disease-free survival was observed with dual therapy of trastuzumab and pertuzumab regimen in neoadjuvant treatment^8,9,22^. The current therapeutic guidelines recommend neoadjuvant treatment with single HER2-targeted therapy of trastuzumab or dual HER2-targeted therapy of trastuzumab and pertuzumab for HER2-positive breast cancer patients in early/locally advanced/inflammatory stages with any tumour sized ≥ 2 cm or with nodal positivity. Dual HER2-targeted therapy of trastuzumab and pertuzumab is especially recommended in high-risk patients with nodal positivity and/or estrogen receptor (ER) negativity^23,24^.

However, the high cost of HER2-targeted agents remains a challenge for low- and middle-income countries (LMICs), and the use of dual HER2-targeted therapy is limited. The economic evaluations on neoadjuvant HER2-targeted therapy in breast cancer treatments are limited and are primarily from high-income countries^25–27^ along with few upper-middle income countries (upper-MICs)^28,29^. Many of them reported that the dual HER2-targeted regimen of trastuzumab and pertuzumab was cost-effective compared to single use of trastuzumab^25–29^. There is no evidence on economic evaluations of these high-cost regimens from lower-MICs that will be beneficial for the reimbursement decisions. Furthermore, Sri Lanka largely provides cancer care though free healthcare provision by the public healthcare system. Trastuzumab is routinely used as HER2-targeted therapy for HER2-positive breast cancer in the public healthcare institutions^7^. While there is limited information on the number of breast cancer patients receiving neoadjuvant HER2 targeted therapy, the percentage of HER2-positive breast cancer patients receiving adjuvant trastuzumab was around 60% in Sri Lanka^30^. However, the use of neoadjuvant systemic therapy in breast cancer is reported to be considerably low^30,31^ Conducting an economic evaluation on the use of dual HER2-targeted medicines for neoadjuvant therapy will provide country-context evidence for consideration to support coverage decisions on these effective high-cost targeted medicines in Sri Lanka. Hence, the objectives of this study were to assess the cost-utility and budget impact of therapy with dual HER2-targeted regimens (trastuzumab and pertuzumab/lapatinib) to single HER2-targeted regimens with trastuzumab for the neoadjuvant treatment of HER2-positive breast cancer in Sri Lanka.

## Methods

The cost-utility analysis was conducted comparing neoadjuvant treatment with dual HER2-targeted regimens to single HER2-targeted regimen using a decision analytical Markov model. The analysis was conducted in public healthcare system and societal perspectives.

### Interventions and comparator

The analysis assessed neoadjuvant therapies of dual HER2-targeted agent combinations, primarily dual therapy with pertuzumab (P) plus trastuzumab (T), with chemotherapy (C) compared to single HER2-targeted agent trastuzumab (T) with chemotherapy (C) as the comparator. An alternative dual therapy of lapatinib (L) plus trastuzumab (T) with C was also considered in the analysis. These dual HER2-targeted treatment regimens in neoadjuvant phase were followed by two sequences of adjuvant therapy (post-surgery) for 1 year, either continuation of same dual HER2-targeted agents or single HER2-targeted therapy of trastuzumab.

As such, the model considered five neoadjuvant-adjuvant treatment strategies; strategy 1: neoadjuvant TC followed by adjuvant T (as the comparator), strategy 2: neoadjuvant PTC followed by adjuvant T, strategy 3: neoadjuvant LTC followed by adjuvant T, strategy 4: neoadjuvant PTC followed by adjuvant PT, and strategy 5: neoadjuvant LTC followed by adjuvant LT. Strategies 2 and 4 were primary intervention regimens with P included dual therapy, and strategies 3 and 5 were alternative intervention regimens with L included dual therapy. Strategy 4 and 5 had the same dual HER2-targeted therapy regimen in neoadjuvant phase continued in the adjuvant phase.

### Target population

The patients included were women with locally advanced, inflammatory or early HER2-positive breast cancer, eligible for neoadjuvant treatment [i.e., tumor diameter ≥ 2 cm or with positive axillary lymph nodes ≥ N1] in accordance with treatment guideline recommendations^23,24,32^. The age of the patients entering the model was 50 years (mean age of breast cancer patients according to the recent surveys conducted in Sri Lanka)^6,7,30,33^.

### Model overview

**Figure 1:**
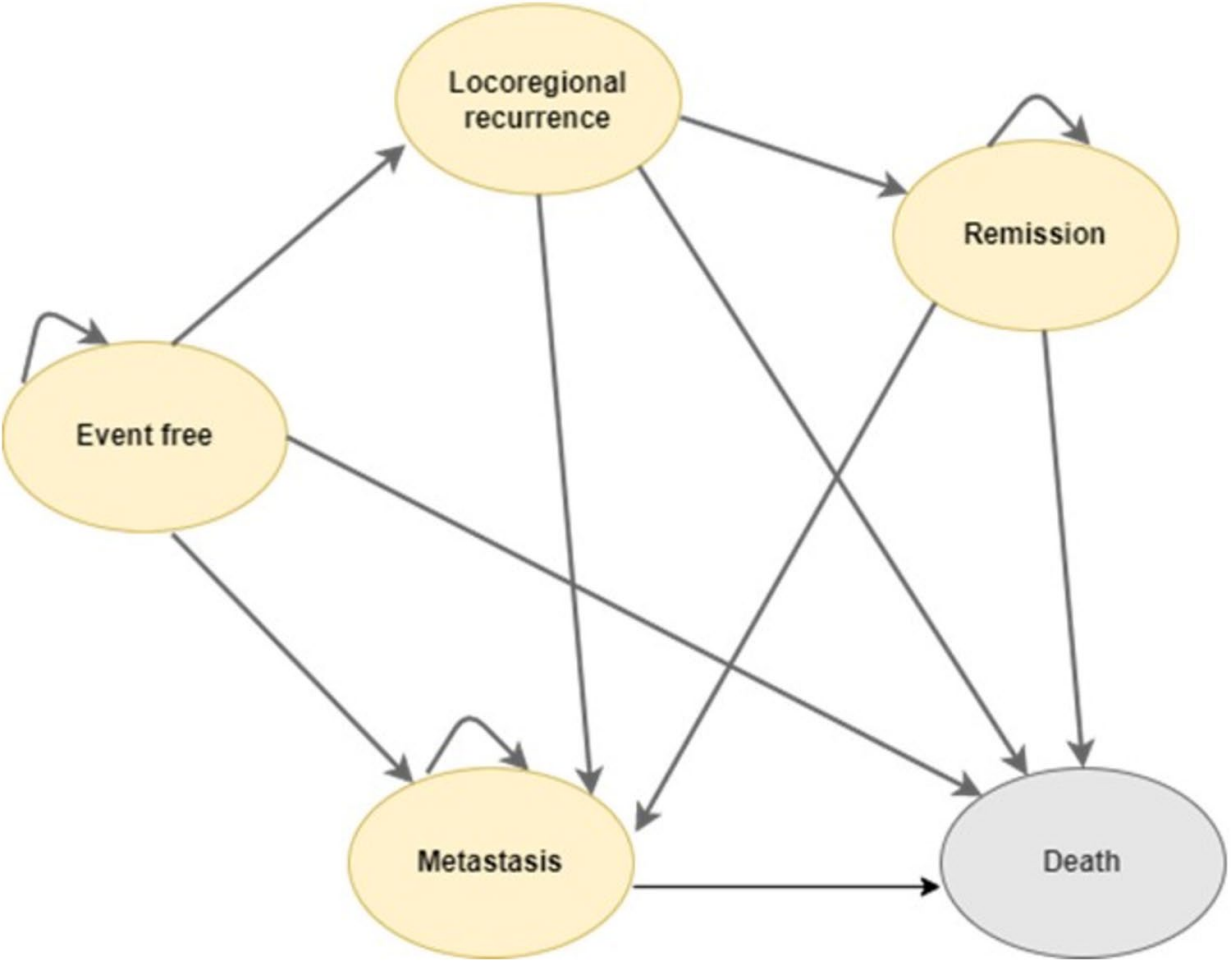
Schematic diagram of the Markov model.

Our cost-utility analysis was conducted using a five-state Markov model including event free, locoregional recurrence, metastasis, remission, and death (Fig. 1). The model was developed considering the six-state model commonly used in previous economic evaluation studies^25,34–36^, and was verified with clinical expert opinion based on the current practice in Sri Lanka. Although the treatment cycle length of neoadjuvant therapy strategies is 3 weeks, the cycle length used in our model was 1 month to be able to capture the effect of treatment. This length was similar to the cycle length that has been used in previous economic evaluations on neoadjuvant HER2 -targeted therapy^25,26,36^. The model was run up to the completion of 100 years of patient’s age to represent lifetime horizon. All costs and health outcomes were discounted at 3% annually^37^.

It was assumed that the chemotherapy regimen used in all arms of interventions were similar and did not have an effect on the final outcomes. All patients who developed locoregional recurrence were to develop it only once, and a failure of treatment or a second locoregional recurrence was assumed to be treated similar to that of distant metastasis. All patients with locoregional recurrence who responded to treatment were moved to remission state, and those who remained in remission state were assumed to be disease free. Death state included both breast cancer related and non-breast cancer related deaths, and death rates were the same as that of general population if disease free.

### Model input parameters

#### Transition probabilities

The transition probabilities for progression to different health states of the model for the treatment cohort of HER2-positive breast cancer patients were estimated from published studies (Table 1)^8,11,36,38–42^. The transition probability from event free (i.e., no recurrences) to events (i.e., locoregional or metastatic recurrence) were estimated using survival data extracted from a published randomized controlled trial based on the comparator regimen^8^. For strategies with dual targeted agents but different adjuvant therapies, survival data from trials with similar respective adjuvant therapy^11,40^ were used for estimation. The age matched all-cause mortality was estimated using the Sri Lankan life tables^43^.

**Table 1:** Parameters of the model and the sampling distributions for the probabilistic sensitivity analysis.

Parameter description	Mean value (Base case analysis)	Standard error	Distribution	References
Transition probabilities (TP)				
TP from event free to event				
Regimens with adjuvant trastuzumab from (0–60 months)				
(strategy 1, strategy 2, strategy 3)	0.01015	0.00102	Beta	Gianni et al. (2016)^8^
Regimen with adjuvant with trastuzumab plus pertuzumab from (0–60 months)				
(strategy 4)				
Time dependent Transition probability				Hurvitz et al. (2019)^40^
Year 1 (0–12 months)	0.00118	0.00012	Beta	
Year 2 (12–24 months)	0.00229	0.00023	Beta	
Year 3 (24–36 months)	0.00220	0.00022	Beta	
Year 4 (36–60 months)	0.00220	0.00022	Beta	
Regimen with adjuvant trastuzumab plus lapatinib from (0–60 months) (strategy 5)	0.00798	0.00080	Beta	Huober et al. (2019)^11^
For all regimens after 60 months	0.01015	0.00102	Beta	Gianni et al. (2016)^8^
Percentage with locoregional recurrence as an event from event free state	25%	0.02500	Beta	Gianni et al. (2016), De Azambuja (2014), NICE (2016)^8,36,38^
Percentage with metastatic recurrence as an event from event free state	75%	0.07500	Beta	
TP from Locoregional recurrence to Metastasis	0.02317	0.00232	Beta	Wang et al. (2018)^41^
TP from locoregional recurrence to Death	0.01064	0.00106	Beta	Wang et al. (2018)^41^
TP from locoregional recurrence to Remission	0.96619	0.09662	Beta	^–^
TP from Remission to Metastasis	0.0076	0.00076	Beta	Hamilton et al. (2015), NICE (2016)^36,42^
TP from Event free state and Remission state to Death (General Population)				
50–54 years	0.00027	0.00003	Beta	Life Tables for Sri Lanka^43^
55–59 years	0.00041	0.00004	Beta	
60–64 years	0.00067	0.00007	Beta	
65–69 years	0.00120	0.00012	Beta	
70–74 years	0.00226	0.00023	Beta	
75–79 years	0.00379	0.00038	Beta	
80–84 years	0.00708	0.00071	Beta	
85 years and above	0.00708	0.00071	Beta	
TP from Metastasis to Death	0.04672	0.00467	Beta	Swain et al. (2020)^39^

Efficacy of intervention Hazard ratio for intervention vs comparator				
	Mean value (Base case analysis)	95%CI	Distribution	
Strategy 2 vs Strategy 1 (comparator)	0.54	0.32–0.91	Log normal	Gunasekara et al. (2022)^21^
Strategy 3 vs Strategy 1 (comparator)	0.85	0.60–1.22	Log normal	
Strategy 4 vs Strategy 1 (comparator)	0.54	0.32–0.91	Log normal	
Strategy 5 vs Strategy 1 (comparator)	0.85	0.60–1.22	Log normal	

Utility parameters				
	Mean value (Base case analysis)	Standard error	Distribution	
Quality of life for Event free state 1st year	0.81	0.0183	Beta	Verill et al. (2020)^44^
Quality of life for Event free state after 1st year	0.85	0.0102	Beta	Li et al. (2019)^45^
Quality of life for Locoregional recurrence state	0.72	0.0198	Beta	Li et al. (2019)^45^
Quality of life for Remission state	0.82	0.0174	Beta	Verill et al. (2020)^44^
Quality of life for Metastasis state	0.70	0.0266	Beta	Verill et al. (2020)^44^

Cost parameters (in LKR)				
	Mean value (Base case analysis)	Standard error	Distribution	
-Direct Medical costs (DMC)				
DMC of Neoadjuvant treatment in Strategy 1 (comparator)	403,337.46	40,333.75	Gamma	Estimation based on cost data from Medical Supplies Division, state hospitals, Ministry of Health, Sri Lanka (2021), Chulasiri et al. (2014) ^46–48^
DMC of Neoadjuvant treatment in Strategy 2	2,591,980.99	259,198.10	Gamma	
DMC of Neoadjuvant treatment in Strategy 3	712,164.10	71,216.41	Gamma	
DMC of Neoadjuvant treatment in Strategy 4	2,591,980.99	259,198.10	Gamma	
DMC of Neoadjuvant treatment in Strategy 5	712,164.10	71,216.41	Gamma	
DMC associated with EF state in Year 1 for Strategy 1 (comparator)	66,740.23	6674.02	Gamma	
DMC associated with EF state in Year 1 for Strategy 2	66,819.49	6681.95	Gamma	
DMC associated with EF state in Year 1 for Strategy 3	66,740.23	6674.02	Gamma	
DMC associated with EF state in Year 1 for Strategy 4	576,956.06	57,695.61	Gamma	
DMC associated with EF state in Year 1 for Strategy 5	157,203.06	15,720.31	Gamma	
DMC associated with EF state in Years 2–5	1097.03	109.70	Gamma	
DMC associated with EF state in Years 6–10	876.94	87.69	Gamma	
DMC associated with EF state after 10 years	404.15	40.42	Gamma	
DMC associated with Locoregional Recurrence state in year 1	307,205.83	30,720.58	Gamma	
DMC associated with Locoregional Recurrence state year 2 onwards	263,124.47	26,312.44	Gamma	
DMC associated with Remission state in year 1	67,429.98	6743.00	Gamma	
DMC associated with Remission state from year 2–5	742.72	74.27	Gamma	
DMC associated with Remission state from year 6–10	1137.50	113.75	Gamma	
DMC associated with Remission state after year 10	664.71	66.47	Gamma	
DMC associated with Metastasis state	111,356.35	11,135.63	Gamma	
-Direct Non-Medical costs (DNMC)				
DNMC in neoadjuvant treatment	61,575.58	6157.56	Gamma	Estimation based on cost data from Chulasiri et al. (2014), Statistics Department, Central Bank of Sri Lanka; (2020)^48,49^
DNMC associated with EF state in Year 1	9,045.57	904.56	Gamma	
DNMC associated with EF state Year 2 onwards	1,768.42	176.84	Gamma	
DNMC associated with Locoregional Recurrence state	103,098.78	10,309.88	Gamma	
DNMC associated with Remission state in Year 1	6,189.48	618.95	Gamma	
DNMC associated with Remission state Year 2 onwards	1,768.42	176.84	Gamma	
DNMC associated with Metastasis state	9,172.73	917.27	Gamma	
Epidemiological parameter				
Prevalence of breast cancer in Sri Lanka (5- year prevalence in all ages)	13,647 prevalent cases		–	Sri Lanka Globocan (2020)^4^
Incidence of breast cancer in Sri Lanka	4447 new cases		–	National cancer control programme (2015)^50^
Percentage of early breast cancer cases in Sri Lanka	66%		–	Wijeratne et al. (2021)^30^
Percentage of HER2 positive breast cancer casesPercentage of HER2 positive breast cancer cases	22%		–	Wijeratne et al. (2021)^30^

*C* Chemotherapy, *DMC* Direct medical cost, *DNMC* Direct non-medical cost, *EF* Event Free, *L* Lapatinib, *P* Pertuzumab, *T* trastuzumab, *TP* transition probability

Strategy 1, Neoadjuvant TC followed by adjuvant T; Strategy 2, Neoadjuvant PTC followed by adjuvant T; Strategy 3, Neoadjuvant LTC followed by adjuvant T; Strategy 4, Neoadjuvant PTC followed by adjuvant PT; Strategy 5, Neoadjuvant LTC followed by adjuvant LT

#### Efficacy

The efficacy parameters used in the model were derived from a systematic review and network meta-analysis (NMA)(Table 1)^21^. Maximum duration for the effect of different neoadjuvant therapies was assumed to be up to 12 years, based on the maximum duration of available survival data for the regimens derived from NMA studies^11,21^.

#### Utilities

The outcome measure for this analysis was quality-adjusted life years (QALYs), which is an estimation of LYs multiplied by utility values. A systematic review was conducted to identify the most appropriate utility values for the respective health states. The values were sourced from health-related quality of life (HRQL) studies that used EuroQol five-dimension with 5-level (EQ-5D-5L) questionnaires reporting utility values among breast cancer patients on HER2-targeted treatment and from Asian countries (Table 1)^44,45^.

#### Costs

The cost parameters consisted of direct medical and direct non-medical costs associated with neoadjuvant treatment phase and each health state for societal perspective, and included only direct medical costs for public healthcare system perspective (Table 1). Indirect costs of patients were excluded in this analysis to prevent double counting. All costs were converted to the year 2021 value using the Consumer Price Index^51,52^ and exchange rate of USD for 2021 (194.78 LKR = 1 USD)^53^.

Direct medical costs comprised initial diagnosis, treatment costs, hospitalization, clinic visits, and follow up costs. The unit costs of hospitalization, clinic visit and diagnostics, procedure (i.e., surgery, radiotherapy) were estimated from the published literature and data available from state sector hospitals^46–48^. The unit costs of medicines were from the database of Sri Lanka medical supplies division (MSD), Ministry of Health. All regimen dosages were based on therapeutic guidelines and dosages used in clinical trials^8,13,17,23,32,38^. The weight and body surface area used for dose calculation of treatment regimens were based on average weight and height of Sri Lankan females extracted from the published literature^54^. Assigning the quantity of units consumed of the cost items was based on the standard care provided for the patients in Sri Lanka^8,13,17,23,30,32,38,55–57^ and clinical expert opinion. Direct non-medical costs included transportation costs, cost of accompanying caregiver, food and other expenses during hospital visits and costs of paid caregiver, which were extracted from the previous costing studies in Sri Lanka^48,49^.

### Statistical analysis

#### Base-case analysis

The incremental cost-effectiveness ratios (ICERs) were estimated for each of the four strategies of dual HER2-targeted therapy regimens compared to the single HER2-targeted therapy regimen. Apart from the traditional ICER calculation, the incremental analysis was employed by arranging all five strategies in ascending order according to the total lifetime costs estimated through the model, and calculating the incremental costs and incremental outcomes relative to the next least-costly strategy.

The value of one-time Gross Domestic Product (GDP) per capita for year 2021 [i.e., LKR758,680 (USD3,815)]^58^ was used as the willingness to pay (WTP) threshold for this study in accordance with the WHO guidance^59,60^.

#### Scenario analysis

This study was conducted in two separate scenarios based on the variation of the unit cost of trastuzumab of the products currently used in the state sector hospitals. Cost parameters estimated for the health states using the highest and lowest trastuzumab unit cost (See Supplementary Table S1), were used as input parameters to assess the cost-effectiveness of each of the regimens.

### Uncertainty analysis

Both one-way and probabilistic sensitivity analyses (PSA) were performed. One-way deterministic sensitivity analysis was performed by changing upper and lower limits of a single parameter while others remain constant, of which the results are presented in the tornado diagram. PSA was performed using Monte Carlo simulation replicated for 1,000 iterations. The cost-effectiveness planes and cost-effectiveness acceptability curves (CEACs) were constructed to show the probability of treatment being the most cost-effective at a given cost-effectiveness threshold. The standard error for the parameters was estimated from 10% of the value for the analysis, except for discounting for costs and outcomes and hazard ratio of efficacy.

### Budget impact analysis

The budget impact for five fiscal years was conducted for the public healthcare system perspective based on the results of the Markov model. The year one included estimated budget for implementation of treatment for new and prevalent cases. The prevalence and incidence of HER2-positive breast cancer was from the published literature and cancer registry data from Sri Lanka^4,30,50^. The budget impact was estimated for coverage of 60% and 20% based on the use of HER2-targeted therapy and neoadjuvant treatment in breast cancer^30^.

### Ethical statement

This study was to assess cost-utility and budget impact of neoadjuvant treatment with HER2 targeted medicines in the treatment of HER2-positive breast cancer in Sri Lanka using retrospective data collected from databases and relevant documents. Therefore, ethical approval for this study was granted as an exemption review and informed consent was waived by the Institutional Review Board of Faculty of Dentistry and Faculty of Pharmacy, Mahidol University, Thailand (COE.No.MU-DT/PY-IRB2022/015.0203), and the Ethics Review Committee of Faculty of Medicine, Kotelawala Defence University, Sri Lanka (RP/2022/09). Furthermore, the permissions were granted from the relevant organizations for access of data. All methods were performed in accordance with the relevant guidelines and regulations.

## Results

### Cost-effectiveness analysis

According to our findings as presented in Table 2, all four intervention strategies (strategies 2–5) of dual HER2-targeted neoadjuvant therapy showed higher outcomes (i.e., LYs, QALYs) and higher total costs than the comparator (strategy 1) with single HER2-targeted therapy T. The incremental QALY gained for strategies 2 and 4 which included PT dual therapy compared to the strategy 1 (neoadjuvant TC followed by adjuvant T) comparator were 1.86 and 3.35 respectively, while the incremental costs of treatments were also comparatively higher for strategies 2 and 4. On average, strategy 4 contributed to the highest outcomes of 12.75 LYs and 10.62 QALYs, and also highest lifetime costs of LKR10,735,582 (or USD55,116) per patient from public healthcare system perspective. The lowest ICER was for strategy 3 (neoadjuvant LTC followed by adjuvant T) with LKR 512,240 or USD2,630 per QALY gained from public healthcare system perspective, and LKR 529,117 or USD2,716 per QALY gained from societal perspective. The second lowest ICER was for strategy 2 (neoadjuvant PTC followed by adjuvant T) with LKR 1,074,254 or USD 5,515 per QALY gained from public healthcare system perspective and LKR 1,090,863 or USD 5,600 per QALY gained from societal perspective.

**Table 2:** Cost-effectiveness and cost-utility of dual HER2-targeted (pertuzumab/lapatinib plus trastuzumab) regimens in base case (deterministic) analysis and incremental analysis.

Regimens	Total LYs		Incremental LYs gained		Total QALYs			Incremental QALYs gained		Cur		Total lifetime costs	Incremental costs			ICER (Cost/LY gained)	ICER (Cost/QALY gained)		Incremental analysis (Cost/QALY gained)	
*Public healthcare system perspective*																				
Strategy 1 (Comparator)	8.87		Reference		7.27			Reference		LKR		2,882,178	Reference			Reference	Reference		Reference	
										USD		14,797								
Strategy 3	9.46		0.59		7.78			0.51		LKR		3,141,642	259,464			442,724	512,240*		512,240*	
										USD		16,129	1,332			2,273	2,630*		2,630*	
Strategy 5	9.96		1.09		8.21			0.94		LKR		4,125,331	1,243,153			1,142,246	1,322,328		2,268,678	
										USD		21,179	6,382			5,864	6,789		11,647	
Strategy 2	11.03		2.16		9.13			1.86		LKR		4,885,031	2,002,853			928,633	1,074,254		821,930	
										USD		25,079	10,283			4,768	5,515		4,220	
Strategy 4	12.75		3.87		10.62			3.35		LKR		10,735,582	7,853,403			2,026,762	2,345,204		3,941,636	
										USD		55,116	40,319			10,406	12,040		20,236	
*Societal perspective*																				
Strategy 1 (Comparator)	8.87		Reference		7.27			Reference		LKR		3,339,498	Reference			Reference	Reference		Reference	
										USD		17,145								
Strategy 3	9.46		0.59		7.78			0.51		LKR		3,607,512	268,013			457,311	529,117		529,117	
										USD		18,521	1,376			2,349	2,716		2,716	
Strategy 5	9.96		1.09		8.21			0.94		LKR		4,598,989	1,259,491			1,157,258	1,339,706		2,286,643	
										USD		23,611	6,466			5,941	6,878		11,739	
Strategy 2	11.03		2.16		9.13			1.86		LKR		5,373,317	2,033,819			942,990	1,090,863		837,756	
										USD		27,586	10,442			4,841	5,600		4,301	
Strategy 4	12.75		3.87		10.62			3.35		LKR		11,249,495	7,909,997			2,041,367	2,362,104		3,958,902	
										USD		57,754	40,609			10,480	12,127		20,325	

*C* Chemotherapy, *L* Lapatinib, *P* Pertuzumab, *T* trastuzumab, *LY* Life Years, *QALY* Quality adjusted Life years.

Strategy 1: Neoadjuvant TC followed by adjuvant T, Strategy 2: Neoadjuvant PTC followed by adjuvant T, Strategy 3: Neoadjuvant LTC followed by adjuvant T, Strategy 4: Neoadjuvant PTC followed by adjuvant PT, Strategy 5: Neoadjuvant LTC followed by adjuvant LT. *Cost effectiveness Threshold (1 GDP per capital of Sri Lanka): LKR 758,680 (3,815 USD).

Overall, the strategies which included dual HER2-targeted therapy in the neoadjuvant phase only (i.e., strategy 2, 3) had lower ICERs compared to the regimens with dual HER2-targeted therapy included in both neoadjuvant and adjuvant phases (i.e., strategy 4, 5).

When every strategy was arranged in ascending order of the total lifetime costs, the only regimen with ICER less than the 1-GDP per capita willingness-to-pay threshold for in Sri Lanka (LKR 758,680 or USD3,815) was strategy 3 compared to the comparator in base case analysis. However, the incremental analysis showed that strategy 2 (Neoadjuvant PTC followed by adjuvant T) had 0.92 incremental QALY gain compared to strategy 5 despite including dual LT therapy in neoadjuvant and adjuvant phases. Strategy 2 compared to strategy 5 also had comparatively lower incremental costs with LKR 837,756 (USD 4,301) cost per QALY gained (Table 2), which was only 11–13% higher than the 1-GDP per capita threshold. The comparison of strategy 4 to strategy 2 also showed higher outcomes (1.48 incremental QALYs). However, the latter was not cost-effective with ICER being LKR 3,958,902 (USD 20,325) per QALY gained in societal perspective (Table 2).

In scenario analysis where cost estimation was based on lowest and highest unit cost of trastuzumab instead of average unit cost used in base case analysis, the scenario 1 (highest unit cost) increased the total lifetime costs by 33%, 19%, 30%, 8% and 23%, and scenario 2 (lowest unit cost) decreased the total lifetime costs only by 10%, 6%, 9%, 3% and 7% in public healthcare system perspective, for strategies 1 to 5 respectively. However, the incremental costs and ICERs for the intervention strategies in both scenarios had minimum changes in comparison to the base-case (See Supplementary Fig. S1).

### Threshold sensitivity analysis

Threshold analysis was conducted for strategies 2 and 4, among three strategies 2, 4 and 5 which were not cost-effective at 1 GDP per capita threshold to assess if these interventions will achieve cost-effectiveness with reduction of treatment costs. Accordingly, a cost reduction of at least 25% of neoadjuvant treatment for strategy 2 was required for it to be cost-effective compared to strategy 1, in the societal perspective at 1 GDP per capita threshold (See Supplementary Figure S2). However, strategy 4 would not be cost-effective even with a 100% reduction of neoadjuvant treatment cost alone, while a 25% cost reduction of neoadjuvant treatment coupled with 70% reduction of adjuvant treatment costs would render the strategy 4 to be cost-effective (See Supplementary Figure S2).

### Deterministic sensitivity analysis (DSA)

According to the one-way DSA, the first 10 parameters that resulted in greater changes of ICERs for strategies 2 to 5 are provided in supplementary material (See Supplementary Figure S3). Among parameters that the model was sensitive in all four strategies included the hazard ratios for efficacy, direct medical costs of neoadjuvant phase, direct medical cost of event free state, discount rates for costs and outcomes and transition probabilities of event-free to event. It is noteworthy, that according to the findings of the one-way DSA, the results were favourable for strategy 1 with single trastuzumab therapy in the comparisons with lapatinib dual therapy (i.e., strategy 3 and 5), as the interventions with lapatinib compared to comparator (strategy 1) had a wide range of hazard ratios with non-significant 95%CIs. At upper limit in efficacy, this resulted in ICERs with negative percentage change for those interventions with lapatinib.

### Probabilistic sensitivity analysis (PSA)

**Figure 2:**
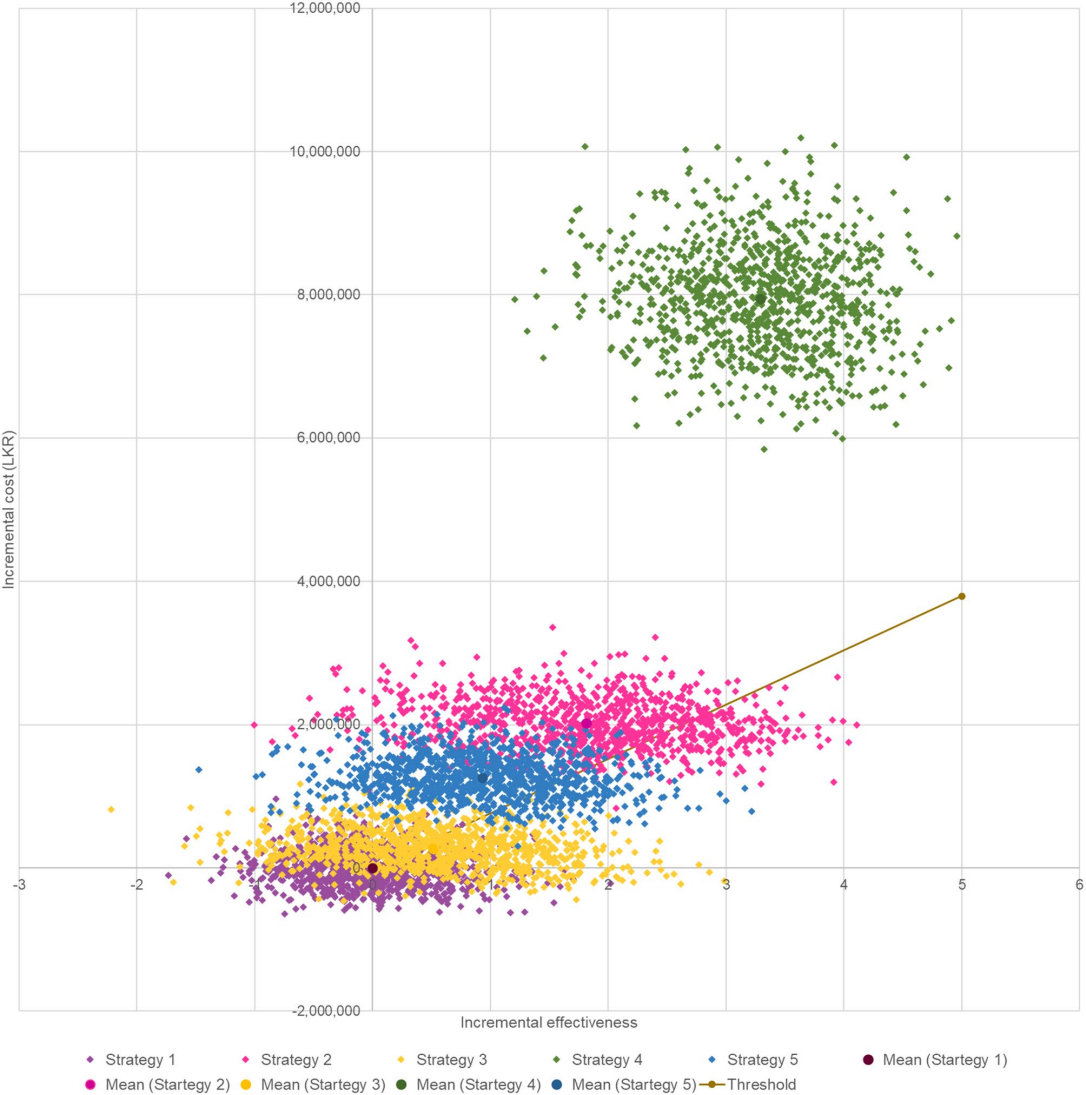
Cost effectiveness plane for societal perspective. C: Chemotherapy, L: Lapatinib, P: Pertuzumab, T: Trastuzumab. Strategy 1: Neoadjuvant TC followed by adjuvant T, Strategy 2: Neoadjuvant PTC followed by adjuvant T, Strategy 3: Neoadjuvant LTC followed by adjuvant T, Strategy 4: Neoadjuvant PTC followed by adjuvant PT, Strategy 5: Neoadjuvant LTC followed by adjuvant LT.

**Figure 3:**
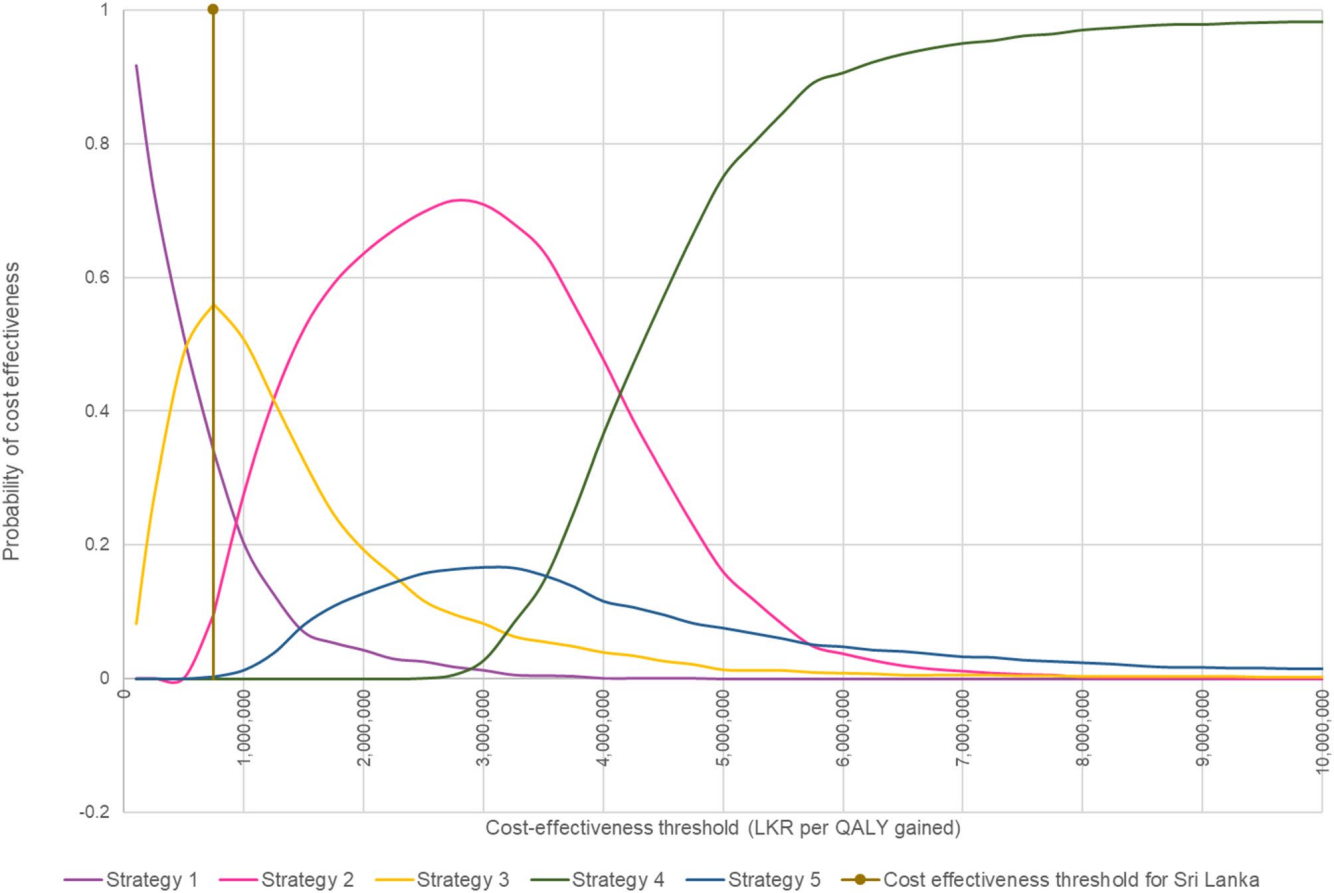
Cost effectiveness acceptability curve for societal perspective. C, Chemotherapy; L, Lapatinib; P, Pertuzumab; T Trastuzumab. Strategy 1: Neoadjuvant TC followed by adjuvant T, Strategy 2: Neoadjuvant PTC followed by adjuvant T, Strategy 3: Neoadjuvant LTC followed by adjuvant T, Strategy 4: Neoadjuvant PTC followed by adjuvant PT, Strategy 5: Neoadjuvant LTC followed by adjuvant LT.

The cost effectiveness results of the PSA were similar to that of the base case analysis and are further illustrated in scatter plot (Fig. 2). While majority of the results for dual HER2-targeted strategies were more effective and costly and fell in northeast quadrant, incremental QALY gain was higher for dual HER2-targeted therapy with PT (i.e., strategies 2 and 4) compared to strategy 1. However, the incremental cost was comparatively higher for strategy 4 compared to all other strategies. As for strategies 3 and 5 with LT, strategy 3 was not dominant compared to strategy 1 single trastuzumab therapy, and both strategies were dominated by strategy 2 with neoadjuvant PT followed by adjuvant T therapy. Furthermore, according to the cost-effectiveness acceptability curves, the probability of cost-effectiveness was 55% and 10% respectively for strategy 3 and strategy 2 at 1 GDP per capita threshold. However, probability of cost-effectiveness increased to 52% for strategy 2 when the threshold increased to 150,000 LKR (Fig. 3).

### Budget impact analysis

In the first year of implementation, the budget impact for the interventions in comparison to the single HER2-targeted therapy regimen strategy 1 were 2.78, 1.25, 7.73 and 2.09-fold higher for strategy 2, 3, 4 and strategy 5 respectively. From year 2 to 5, the budget was around twofold higher for strategy 2 compared to strategy 1 regimen (Table 3). The first-year incremental budget was LKR 161 million (USD 0.82 million) and LKR 482 million (USD 2.47 million) for strategy 3 and LKR 1145 million (USD 5.88 million) and LKR 3,435 million (USD 17.64 million) for strategy 2, at 20% coverage and 60% coverage respectively (Table 3).

**Table 3:** Budget impact of dual HER2-targeted therapy regimens.

Intervention	Cur	Year 1	Year 2	Year 3	Year 4	Year 5
Incremental budget (in millions) at 60% coverage						
Strategy 1 (Comparator)	Reference					
Strategy 2	LKR	3,435	761	728	706	691
	USD	17.64	3.91	3.74	3.62	3.55
Strategy 3	LKR	482	92	83	77	73
	USD	2.47	0.47	0.42	0.39	0.38
Strategy 4	LKR	13,011	3024	2952	2897	2854
	USD	66.80	15.53	15.15	14.87	14.65
Strategy 5	LKR	2,110	459	437	422	413
	USD	10.83	2.36	2.24	2.17	2.12
Incremental budget (in millions) at 20% coverage						
Strategy 1 (Comparator)	Reference					
Strategy 2	LKR	1,145	254	243	235	230
	USD	5.88	1.30	1.25	1.21	1.18
Strategy 3	LKR	161	31	28	26	24
	USD	0.82	0.16	0.14	0.13	0.13
Strategy 4	LKR	4,337	1,008	984	966	951
	USD	22.27	5.18	5.05	4.96	4.88
Strategy 5	LKR	703	153	146	141	138
	USD	3.61	0.79	0.75	0.72	0.71

Ratio of budget compared to strategy 1 (comparator)						
Intervention	Ratio of budget					
	Year 1	Year 2	Year 3	Year 4	Year 5	
Strategy 2	2.78	2.11	1.93	1.83	1.76	
Strategy 3	1.25	1.13	1.11	1.09	1.08	
Strategy 4	7.73	5.40	4.78	4.40	4.13	
Strategy 5	2.09	1.67	1.56	1.49	1.45	

*C* Chemotherapy, *L* Lapatinib, *P* Pertuzumab, *T* trastuzumab.

Strategy 1: Neoadjuvant TC followed by adjuvant T, Strategy 2: Neoadjuvant PTC followed by adjuvant T, Strategy 3: Neoadjuvant LTC followed by adjuvant T, Strategy 4: Neoadjuvant PTC followed by adjuvant PT, Strategy 5: Neoadjuvant LTC followed by adjuvant LT.

## Discussion

Our study explored the cost-effectiveness and budget impact of neoadjuvant treatment by adding dual HER2-targeted agents to single HER2-targeted therapy with trastuzumab (T) in the neoadjuvant treatment of early HER2-positive breast cancer in Sri Lanka. Among the dual HER2-targeted therapy strategies neoadjuvant PTC followed by adjuvant T regimen (strategy 2) had higher incremental outcome gain (QALYs, LYs) compared to other strategies without pertuzumab. However, the strategy 2 would be cost-effective compared to single trastuzumab therapy, only if the cost-effectiveness threshold (CE threshold) was two times GDP per capita for Sri Lanka. The findings from threshold analysis indicated that a 25% cost reduction of neoadjuvant treatment could make the neoadjuvant PTC followed by adjuvant T (strategy 2) to be cost-effective at a 1 GDP per capita CE threshold for Sri Lanka in the societal perspective. Even though strategy 4 (neoadjuvant PTC followed by adjuvant PT) had highest outcomes, the incremental analysis revealed that comparison of strategy 2 to strategy 4 had the highest cost per QALY gained when comparing dual HER2-targeted regimen strategies to each other. Furthermore, dual HER2-targeted regimens provided in both neoadjuvant and adjuvant phases tended to be not cost-effective. Based on new long-term survival and meta-analyses results with LT therapy^11,21,61^, the cost-effectiveness of alternative lapatinib included regimens were assessed in the study. The findings present that neoadjuvant LTC followed by adjuvant T (strategy 3) had lowest ICER and was below 1 GDP per capita CE threshold. However, the sensitivity analysis revealed that due to the uncertainty of efficacy of lapatinib included regimens, the results would favour the strategy 1 (neoadjuvant TC followed by adjuvant T).

Our study also found that the budget impact to the public healthcare system of Sri Lanka by inclusion of dual HER2-targeted therapy in the neoadjuvant phase including strategy 2 and strategy 3 would be relatively higher compared to the single HER2-targeted therapy neoadjuvant TC followed by adjuvant T strategy.

To our knowledge, this is the first economic evaluation study in Sri Lanka to compare costs and utilities of neoadjuvant therapy with dual HER2-targeted regimens for HER2-positive breast cancer patients. During the time of this analysis, very few economic evaluations explored dual HER2-targeted therapy neoadjuvant regimens in breast cancer from lower-MICs context. Our economic evaluation was also conducted using pooled efficacy with long term survival data for neoadjuvant regimens, including for neoadjuvant treatment with HER2-targeted therapy exploring the regimens that contained lapatinib, and continuation of same dual HER2-targeted regimens in neoadjuvant and adjuvant phases.

Previous economic evaluation studies conducted in high income countries (e.g., Canada and USA), and in upper-MICs (e.g., China) reported that the regimen of neoadjuvant PTC followed by adjuvant T (as strategy 2 of our study) tended to be cost-effective^26–29,35,62^ in their country contexts, where their WTP or CE thresholds were much higher than the threshold applied for Sri Lanka. However, the studies also reported a higher ICER value and higher costs compared to our findings^25,26,28,29,35^. It was noted that previous studies in contrast to our study also used efficacy parameters of the strategies directly based on the clinical trial^13^ results. The differences in reported total health outcome gain may have been due to limitations in survival and follow up data and effects of crossover. Furthermore, some previous economic evaluations had included novel treatments such as trastuzumab emtansine (T-DM1) and T-DM1 plus pertuzumab for the adjuvant therapy treatment sequences^27,62^, which was not available in Sri Lanka at the time of the analysis. Additionally, few studies explored the cost-effectiveness for hormone receptor negative populations^27,62^.

Results from uncertainty analysis were sensitive to direct medical costs of neoadjuvant and adjuvant treatment phases. In our study, we calculated cost parameters by using multiple sources representing standard costs and utilization of services, which may have caused an underestimation of cost. Hence, more comprehensive costing studies on the public healthcare system in Sri Lanka will be beneficial to provide more up-to-date information for economic evaluations. However, the model showed relatively minimal sensitivity to many parameters proving the robustness of the results.

Our study findings demonstrate that, even though PT dual therapy as neoadjuvant treatment is a better choice compared to single HER2-targeted therapy, the ICER is considerably higher for the regimen that results in higher budget impact. Hence, it could be challenging for lower-MICs such as Sri Lanka to enhance funding for the therapy in the public healthcare system. Although price reductions seem to be an option for the possible use of PT dual therapy in neoadjuvant phase alone, variety of negotiation approaches could be considered for high-cost interventions (i.e., cancer therapy), such as managed entry agreements, risk-sharing agreements, or special access scheme for cancer treatments^63^. Moreover, compulsory and voluntary licensing are other options that are also being implemented for certain cancer medicines especially to improve the access of cancer treatment in lower-MICs^64,65^.

In consideration of the downturn of Sri Lankan economy in year 2022, the year-on-year headline inflation increased by over 60%^66^ by the end of 2022 from 2021 and the national consumer price index increased by 56%^67^ accompanied by the depreciation of the Sri Lankan Rupee. This likely impacted the rise of direct medical and non-medical costs. However, the cost-effectiveness of interventions in this study was assessed based on costs estimated for year 2021. Hence, considering the final results based on USD values will be more appropriate to have an understanding on the present costs in the current year for the Sri Lankan setting.

Some of the limitations experienced in our study were that firstly, novel therapies such as T-DM1, were not included as the analysis focused on dual therapies registered in Sri Lanka at the time of the study. Secondly, limitations were present in availability of evidence on effectiveness and survival along with lesser duration of follow-up for some dual therapy regimens. However, a systematic review and network meta-analysis with mixed effects parametric analysis^21^ was performed to synthesize up to date evidence of treatment efficacy, and best available evidence were used for clinical parameter estimation. Thirdly, there were limitations in the data sources for the cost parameter estimations. However, the cost estimations of this study were based on the standard costs for treatment in the country which was in-line with the current clinical practice in Sri Lanka. Additionally, the uncertainty of parameters was addressed by conducting one-way and probabilistic sensitivity analyses and the results were not considerably different in terms of the final conclusions. Nevertheless, the use and the generalizability of our findings should be done with caution due to the uncertainty of some parameters used in the model. Lastly, Sri Lanka currently does not have a CE threshold set for economic evaluations, and thus 1 GDP per capita of Sri Lanka (2021) was used as the threshold for this analysis. However, this may not be adequate to reflect the WTP for a QALY gained of the country.

## Conclusions

The dual HER2-targeted regimens that included pertuzumab showed higher health outcomes compared to single trastuzumab regimen; nevertheless, in order for the regimen of neoadjuvant PTC followed by adjuvant T to be cost-effective for the Sri Lankan public healthcare system, a cost reduction of the neoadjuvant therapy should be arranged.

### Supplementary Information

Supplementary Information.

## Data Availability

Data is provided within the manuscript or supplementary information files.
