# Supplementary material for: Cost-utility and budget impact analysis of neoadjuvant dual HER2 targeted therapy for HER2-positive breast cancer in Sri Lanka
Source: Sci Rep. 2024 Jul 20;14:16736. doi: 10.1038/s41598-024-67598-2 (PMC11271297; doi:10.1038/s41598-024-67598-2)
Supplement: Supplementary file 1 — Supplementary Information. [file 41598_2024_67598_MOESM1_ESM.pdf]

Cost-utility and budget impact analysis of neoadjuvant dual HER2 targeted therapy for HER2-positive breast cancer in Sri Lanka

Agampodi Danushi Mendis Gunasekara[1,2], Sitaporn Youngkong[1,3*], Thunyarat Anothaisintawee[1,4], Thitiya Dejthevaporn[5], Rohini Fernandopulle[2], Usa Chaikledkaew[1,3]

[1]Mahidol University Health Technology Assessment (MUHTA) Graduate Program, Mahidol University, Bangkok, Thailand.

[2]Department of Paraclinical Sciences, Faculty of Medicine, General Sir John Kotelawala Defence University, Ratmalana, Sri Lanka.

[3]Social and Administrative Pharmacy Division, Department of Pharmacy, Faculty of Pharmacy, Mahidol University, Bangkok, Thailand.

[4]Department of Family Medicine, Faculty of Medicine, Ramathibodi Hospital, Mahidol University, Bangkok, Thailand.

[5]Division of Medical Oncology, Ramathibodi Hospital, Mahidol University, Bangkok, Thailand.

***** Corresponding author:

Sitaporn Youngkong, Ph.D.

E-mail: sitaporn.you@mahidol.edu

Social and Administrative Pharmacy Division, Department of Pharmacy, Faculty of Pharmacy, Mahidol University

447 Sri-Ayudhaya Rd., Phayathai, Ratchathevi, Bangkok 10400, Thailand

Supplementary Table S1: Cost Parameters (in LKR) of the scenario 1 and scenario 2 based on trastuzumab unit cost

Cost parameter	Scenario 1 Highest unit cost	Scenario 2 Lowest unit cost
DMC of Neoadjuvant treatment in Strategy 1 (comparator)	549,559.36	359,308.38
DMC of Neoadjuvant treatment in Strategy 2	2,738,202.88	2,547,951.90
DMC of Neoadjuvant treatment in Strategy 3	858,386.00	668,135.01
DMC of Neoadjuvant treatment in Strategy 4	2,738,202.88	2,547,951.90
DMC of Neoadjuvant treatment in Strategy 5	858,386.00	668,135.01
DMC associated with EF state in Year 1 for Strategy 1 (comparator)	106,107.66	54,886.24
DMC associated with EF state in Year 1 for Strategy 2	106,186.92	54,965.51
DMC associated with EF state in Year 1 for Strategy 3	106,107.66	54,886.24
DMC associated with EF state in Year 1 for Strategy 4	616,323.49	565,102.07
DMC associated with EF state in Year 1 for Strategy 5	196,570.49	145,349.07
DMC associated with EF state in Years 2-5	1,097.03	1,097.03
DMC associated with EF state in Years 6-10	876.94	876.94
DMC associated with EF state after 10 years	404.15	404.15
DMC associated with Locoregional Recurrence state in year 1	340,949.34	297,045.27
DMC associated with Locoregional Recurrence state year 2 onwards	263,124.47	263,124.47
DMC associated with Remission state in year 1	106,797.42	55,576.00
DMC associated with Remission state from year 2-5	742.72	742.72
DMC associated with Remission state from year 6-10	1,137.50	1,137.50
DMC associated with Remission state after year 10	664.71	664.71
DMC associated with Metastasis state	137,460.70	103,496.03

C, Chemotherapy; DMC, Direct medical costs; DNMC, Direct non medical costs; EF, Event free; L, Lapatinib; P: Pertuzumab; T Trastuzumab,
Strategy 1: Neoadjuvant TC followed by adjuvant T, Strategy 2: Neoadjuvant PTC followed by adjuvant T, Strategy 3: Neoadjuvant LTC followed by adjuvant T, Strategy 4: Neoadjuvant PTC followed by adjuvant PT, Strategy 5: Neoadjuvant LTC followed by adjuvant LT

Supplementary Figure S1: Total lifetime cost and Incremental cost-effectiveness ratio of scenario 1 and 2 compared to base case in public healthcare system perspective.

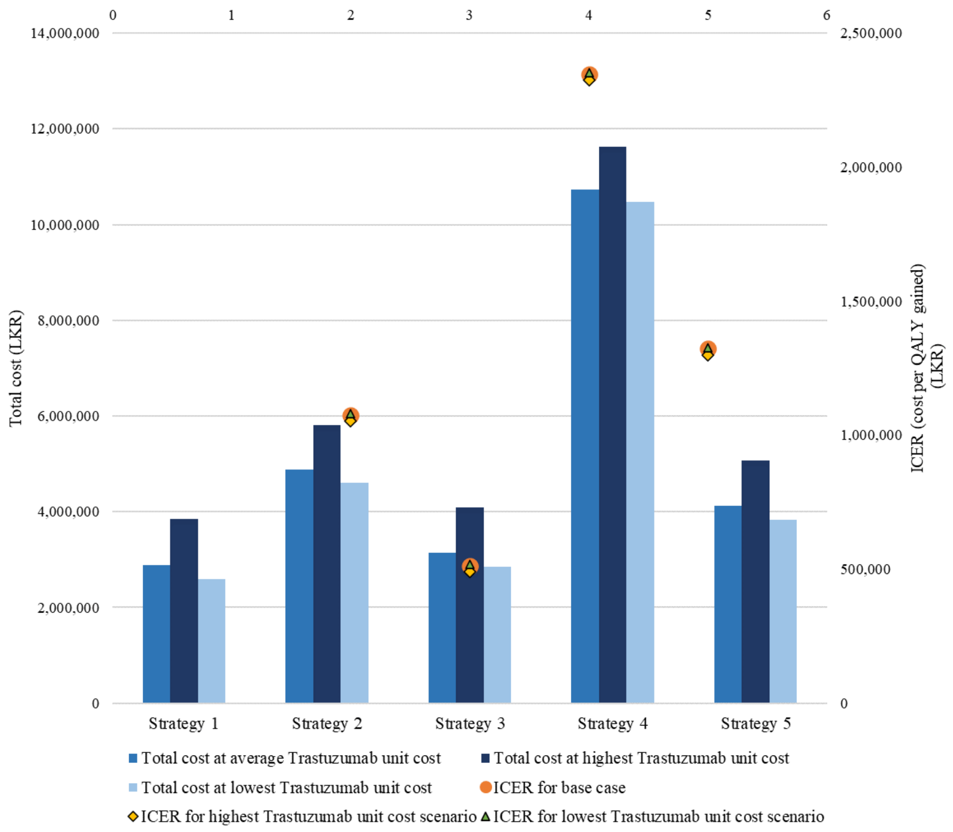

Strategy 1: Neoadjuvant TC followed by adjuvant T, Strategy 2: Neoadjuvant PTC followed by adjuvant T, Strategy 3: Neoadjuvant LTC followed by adjuvant T, Strategy 4: Neoadjuvant PTC followed by adjuvant PT, Strategy 5 : Neoadjuvant LTC followed by adjuvant LT
C, Chemotherapy; L, Lapatinib; P, Pertuzumab; T Trastuzumab

Supplementary Figure S2: Threshold analysis A. Strategy 2, B. Strategy 4

A. **B.**

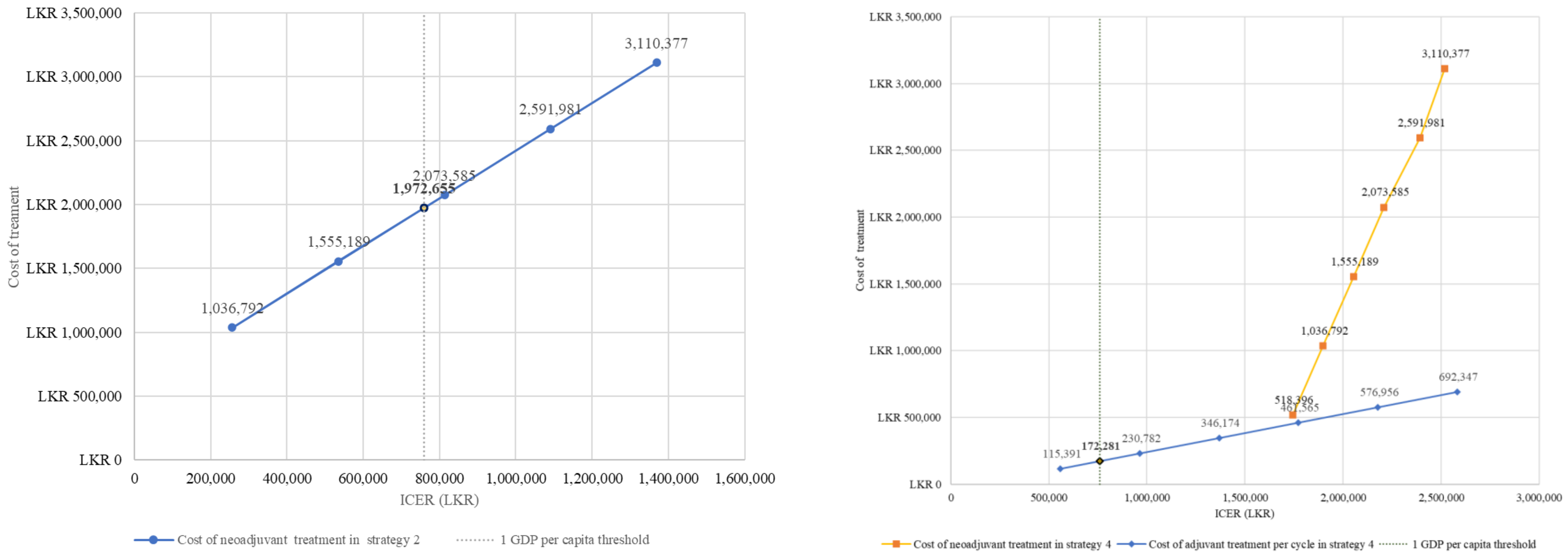

Strategy 2: Neoadjuvant PTC followed by adjuvant T, Strategy 4: Neoadjuvant PTC followed by adjuvant PT
C, Chemotherapy; P: Pertuzumab; T, Trastuzumab

Supplementary Figure S3: Tornado diagrams of Strategy 2, 3, 4, 5 vs Strategy 1(comparator) in Societal perspective. A. Strategy 2 vs Strategy 1, B. Strategy 3 vs Strategy 1, C. Strategy 4 vs Strategy 1, D. Strategy 5 vs Strategy 1

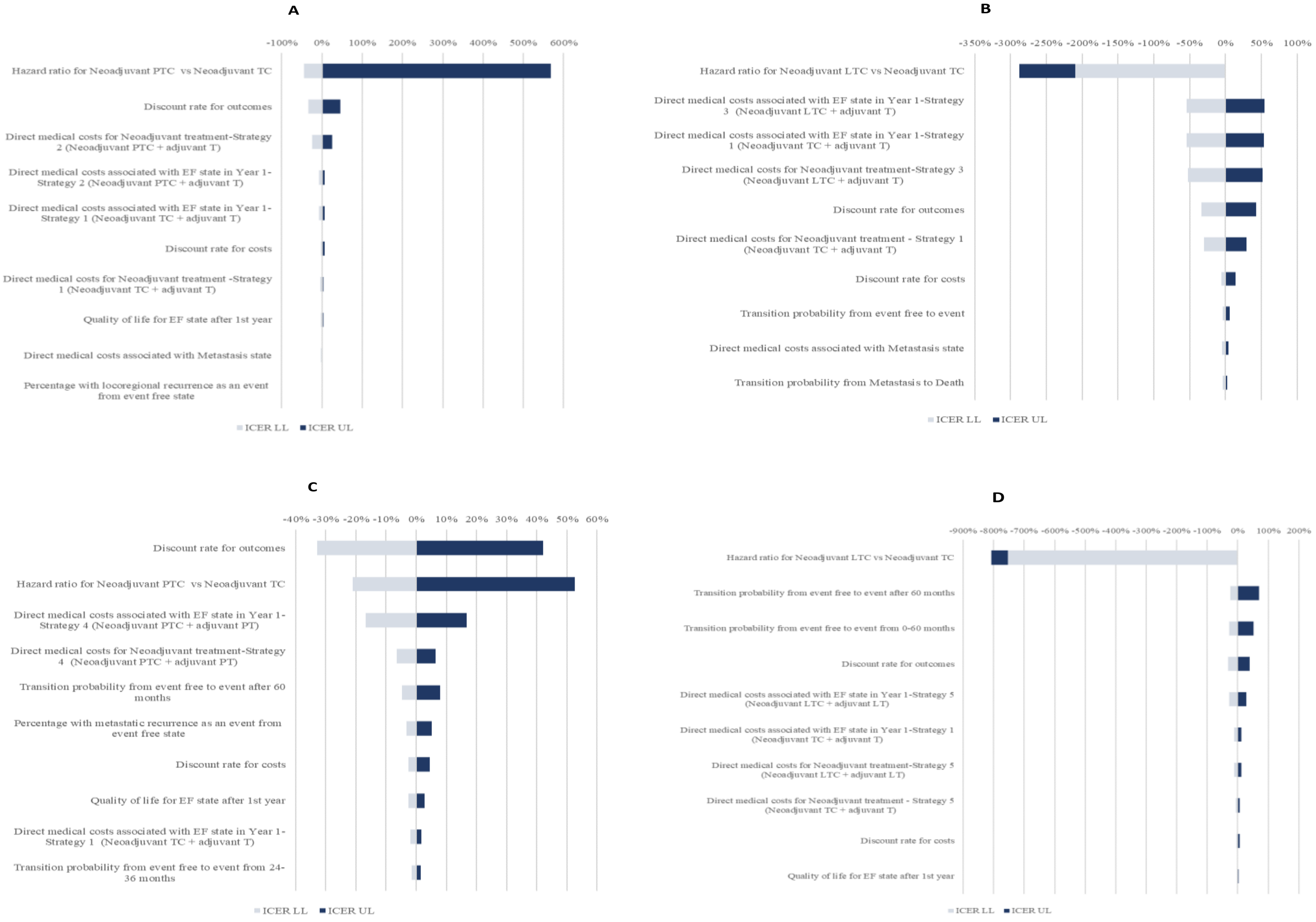

Strategy 1: Neoadjuvant TC followed by adjuvant T, Strategy 2: Neoadjuvant PTC followed by adjuvant T, Strategy 3: Neoadjuvant LTC followed by adjuvant T, Strategy 4: Neoadjuvant PTC followed by adjuvant PT, Strategy 5: Neoadjuvant LTC followed by adjuvant LT
C, Chemotherapy; L, Lapatinib; P: Pertuzumab; T Trastuzumab
